# Survival by Depth of Response and Efficacy by International Metastatic Renal Cell Carcinoma Database Consortium Subgroup with Lenvatinib Plus Pembrolizumab Versus Sunitinib in Advanced Renal Cell Carcinoma: Analysis of the Phase 3 Randomized CLEAR Study

**DOI:** 10.1016/j.euo.2023.01.010

**Published:** 2023-01-29

**Authors:** Viktor Grünwald, Thomas Powles, Evgeny Kopyltsov, Vadim Kozlov, Teresa Alonso-Gordoa, Masatoshi Eto, Thomas Hutson, Robert Motzer, Eric Winquist, Pablo Maroto, Bhumsuk Keam, Giuseppe Procopio, Shirley Wong, Bohuslav Melichar, Frederic Rolland, Mototsugu Oya, Karla Rodriguez-Lopez, Kenichi Saito, Jodi McKenzie, Camillo Porta

**Affiliations:** a Interdisciplinary Genitourinary Oncology, Clinic for Urology, Clinic for Medical Oncology, University Hospital Essen, Essen, Germany; b The Royal Free NHS Trust, London, England, UK; c State Institution of Healthcare ‘‘Regional Clinical Oncology Dispensary’’, Omsk, Russia; d State Budgetary Health Care Institution ‘‘Novosibirsk Regional Clinical Oncology Dispensary’’, Novosibirsk, Russia; e Hospital Universitario Ramón y Cajal, Madrid, Spain;; f Kyushu University, Fukuoka, Japan; g Texas Oncology, Dallas, TX, USA; h Memorial Sloan Kettering Cancer Center, New York, NY, USA; i University of Western Ontario, London, Ontario, Canada; j Hospital de la Santa Creu i Sant Pau, Barcelona, Spain; k Seoul National University Hospital, Seoul, Republic of Korea; l Fondazione IRCCS Istituto Nazionale dei Tumori di Milano, Italy; m Western Health, VIC, Australia; n Palacký University Medical School and Teaching Hospital, Olomouc, Czech Republic; o Centre René Gauducheau Centre de Lutte Contre Le Cancer Nantes, Saint-Herblain, France; p Keio University School of Medicine, Tokyo, Japan; q Merck & Co., Inc, Rahway, NJ, USA; r Eisai Inc, Nutley, NJ, USA; s University of Bari ‘A. Moro’, Bari, Italy

**Keywords:** Lenvatinib, Pembrolizumab, Sunitinib, Depth of response, Renal cell carcinoma

## Abstract

**Background:**

The extent of tumor shrinkage has been deemed a predictor of survival for advanced/metastatic renal cell carcinoma (RCC), a disease with historically poor survival.

**Objective:**

To perform an exploratory analysis of overall survival (OS) by tumor response by 6 mo, and to assess the efficacy and survival outcomes in specific subgroups.

**Design, setting, and participants:**

CLEAR was an open-label, multicenter, randomized, phase 3 trial of first-line treatment of advanced clear cell RCC.

**Intervention:**

Patients were randomized 1:1:1 to lenvatinib 20 mg orally daily with pembrolizumab 200 mg intravenously once every 3 wk, lenvatinib plus everolimus (not included in this analysis), or sunitinib 50 mg orally daily for 4 wk on treatment/2 wk of no treatment.

**Outcome measurements and statistical analysis:**

Landmark analyses were conducted to assess the association of OS with tumor shrinkage and progressive disease status by 6 mo. Progression-free survival, duration of response, and objective response rate (ORR) were analyzed by the International Metastatic Renal Cell Carcinoma Database Consortium (IMDC) risk subgroup and by the presence of target kidney lesions. Efficacy was assessed by an independent review committee as per Response Evaluation Criteria in Solid Tumors version 1.1.

**Results and limitations:**

Landmark analyses by tumor shrinkage showed that patients enrolled to lenvatinib plus pembrolizumab arm with a confirmed complete response or >75% target-lesion reduction by 6 mo had a 24-mo OS probability of ≥91.7%. A landmark analysis by disease progression showed that patients with no progression by 6 mo had lower probabilities of death in both arms. Patients with an IMDC risk classification of intermediate/poor had longer median progression-free survival (22.1 vs 5.9 mo) and a higher ORR (72.4% vs 28.8%) with lenvatinib plus pembrolizumab versus sunitinib. Similarly, results favored lenvatinib plus pembrolizumab in IMDC-favorable patients and those with/without target kidney lesions. Limitations of the study are that results were exploratory and not powered/stratified.

**Conclusions:**

Lenvatinib plus pembrolizumab showed improved efficacy versus sunitinib for patients with advanced RCC; landmark analyses showed that tumor response by 6 mo correlated with longer OS.

**Patient summary:**

In this report of the CLEAR trial, we explored the survival of patients with advanced renal cell carcinoma by assessing how well they initially responded to treatment. We also explored how certain groups of patients responded to treatment overall. Patients were assigned to cycles of either lenvatinib 20 mg daily plus pembrolizumab 200 mg every 3 wk or sunitinib 50 mg daily for 4 wk (followed by a 2-wk break). Patients who either had a ‘‘complete response’’ or had their tumors shrunk by >75% within 6 mo after starting treatment with lenvatinib plus pembrolizumab had better survival than those with less tumor reduction by 6 mo. Additionally, patients who had more severe disease (as per the International Metastatic Renal Cell Carcinoma Database Consortium) at the start of study treatment survived for longer without disease progression with lenvatinib plus pembrolizumab than with sunitinib.

## 1. Introduction

Kidney cancer is one of the most common cancer types in the developed world, accounting for about 4% of new cancer cases in the USA in 2021 and 3.2% of new cases in Europe in 2020 [1,2], and the predominant histology is clear cell renal cell carcinoma (RCC) [3]. Approximately one-third of RCC cases are diagnosed as advanced/metastatic RCC, which historically has a poor survival probability (≤12% at 5 yr) [3,4]. Despite efficacy of first-line vascular endothelial growth factor receptor tyrosine kinase inhibitors for advanced RCC, the development of treatment resistance remains a barrier to long-term survival [5,6]. Combination regimens of immune checkpoint inhibitors (ICIs; nivolumab plus ipilimumab) and ICIs plus kinase inhibitors (pembrolizumab [or avelumab] plus axitinib, pembrolizumab plus lenvatinib, and nivolumab plus cabozantinib) have provided better outcomes than sunitinib monotherapy for patients with advanced/metastatic RCC [7–12]. Recently, these ICI-based combination regimens have become the standard of care [13–15].

Lenvatinib is a multitargeted tyrosine kinase inhibitor that has shown efficacy in patients with advanced RCC as monotherapy or when combined with everolimus after one prior antiangiogenic therapy [16,17]. Pembrolizumab has shown promise as first-line monotherapy for advanced RCC [18,19]. Lenvatinib plus pembrolizumab has also shown efficacy as later-line therapy in a phase 1b/2 study of patients with metastatic RCC (study 111/KEYNOTE-146) [20].

CLEAR (study 307/KEYNOTE-581) was a phase 3 multicenter, open-label, randomized trial that compared the efficacy and safety of lenvatinib plus pembrolizumab or everolimus versus sunitinib alone as first-line treatment for patients with advanced RCC [9]. In this study, the combination of lenvatinib plus pembrolizumab demonstrated significant clinical benefit; clinically meaningful improvements in progression-free survival (PFS), overall survival (OS), and objective response rate (ORR) compared with sunitinib were observed. Of note, the number of patients achieving a complete response (CR) was also noticeably greater with lenvatinib plus pembrolizumab than that with sunitinib.

Given that the extent of tumor shrinkage has been shown to be prognostic in patients with metastatic RCC [21], we conducted landmark analyses to explore the association between OS and depth of tumor response (maximum reduction from baseline in sums of diameters of target lesions), and the association between OS and confirmed CR based on the best response. We also explored efficacy according to select patient subgroups (ie, the International Metastatic Renal Cell Carcinoma Database Consortium [IMDC] risk group and the presence or absence of a target kidney lesion at baseline) among patients randomly assigned to receive lenvatinib plus pembrolizumab versus sunitinib.

## 2. Patients and methods

### 2.1. Study design and patients

The design and protocol of CLEAR (study 307/KEYNOTE 581) have been reported [9]. Briefly, patients were randomly assigned (1:1:1) to receive lenvatinib 20 mg orally once daily plus pembrolizumab 200 mg intravenously every 3 wk, or lenvatinib 18 mg plus everolimus 5 mg orally once daily, or sunitinib 50 mg orally once daily (4 wk on/2 wk off). Eligible patients had previously untreated, advanced RCC with a clear cell component and at least one measurable lesion according to Response Evaluation Criteria in Solid Tumors version 1.1 (RECIST v1.1), a Karnofsky performance status score of ≥70%, adequately controlled blood pressure, and adequate organ function. Randomization was stratified by geographic region (region 1: Western Europe and North America, or region 2: the rest of the world) and Memorial Sloan Kettering Cancer Center (MSKCC) prognostic risk group (favorable, intermediate, or poor).

### 2.2. Endpoints of CLEAR (study 307/KEYNOTE 581)

The primary endpoint of CLEAR was PFS assessed by an independent review committee (IRC) as per RECIST v1.1. Additional endpoints including OS, ORR, health-related quality of life, and safety have been reported [9,22].

### 2.3. Exploratory analyses

Post hoc 6- and 9-mo landmark analyses assessed the association between tumor shrinkage and OS; a 6-mo landmark analysis also assessed the association between progressive disease and OS. For the landmark analyses, survival following landmark time points was assessed, and estimates in patients who were at risk at the landmark time point were presented as the time from randomization. Additionally, degree of tumor shrinkage in the landmark analyses represented the extent of shrinkage prior to the respective landmark time. Subgroup analyses of PFS, ORR, duration of response (DOR), and OS were assessed based on the IMDC risk group (intermediate/poor or favorable) and the presence or absence of a target kidney lesion (identified by IRC) at baseline in the lenvatinib plus pembrolizumab and sunitinib treatment arms. Notably, IMDC risk group was used in this study over MSKCC so that results could be contextualized with existing studies of immune-based combinations for RCC [7,8,15]. The maximum tumor shrinkage from baseline in target kidney lesions was assessed in patients without prior nephrectomy in the lenvatinib plus pembrolizumab and sunitinib treatment arms. Finally, a post hoc exploratory analysis was performed to characterize patients who had either a confirmed CR or a near CR (>75% reduction in tumor size). Efficacy analyses, including assessments of response and PFS, were assessed by IRC as per RECIST v1.1. Efficacy analyses were performed in the intention–to-treat population, landmark analyses were assessed in patients alive at the specified time point, and maximum tumor shrinkage included patients with baseline and one or more postbaseline tumor assessments.

### 2.4. Statistical analyses

The same statistical methods used for the efficacy analyses in CLEAR [9] were applied for the post hoc analyses. Additional statistical analysis details are available in the Supplementary material.

## 3. Results

### 3.1. Baseline demographic and clinical characteristics of patients

From October 13, 2016 to July 24, 2019, 1417 patients were screened and 1069 were randomly assigned to one of three treatment arms in the CLEAR trial; the CONSORT diagram has previously been published [9]. Of these 1069 patients, 355 were assigned to lenvatinib plus pembrolizumab and 357 were assigned to sunitinib. Baseline characteristics of patients in CLEAR, including the prevalence, number, and size of target kidney lesions, were similar and well balanced between the treatment arms (Supplementary Table 1). The median follow-up time in this study was 26.6 mo [9].

### 3.2. Landmark analyses of OS

**Fig. 1 –:**
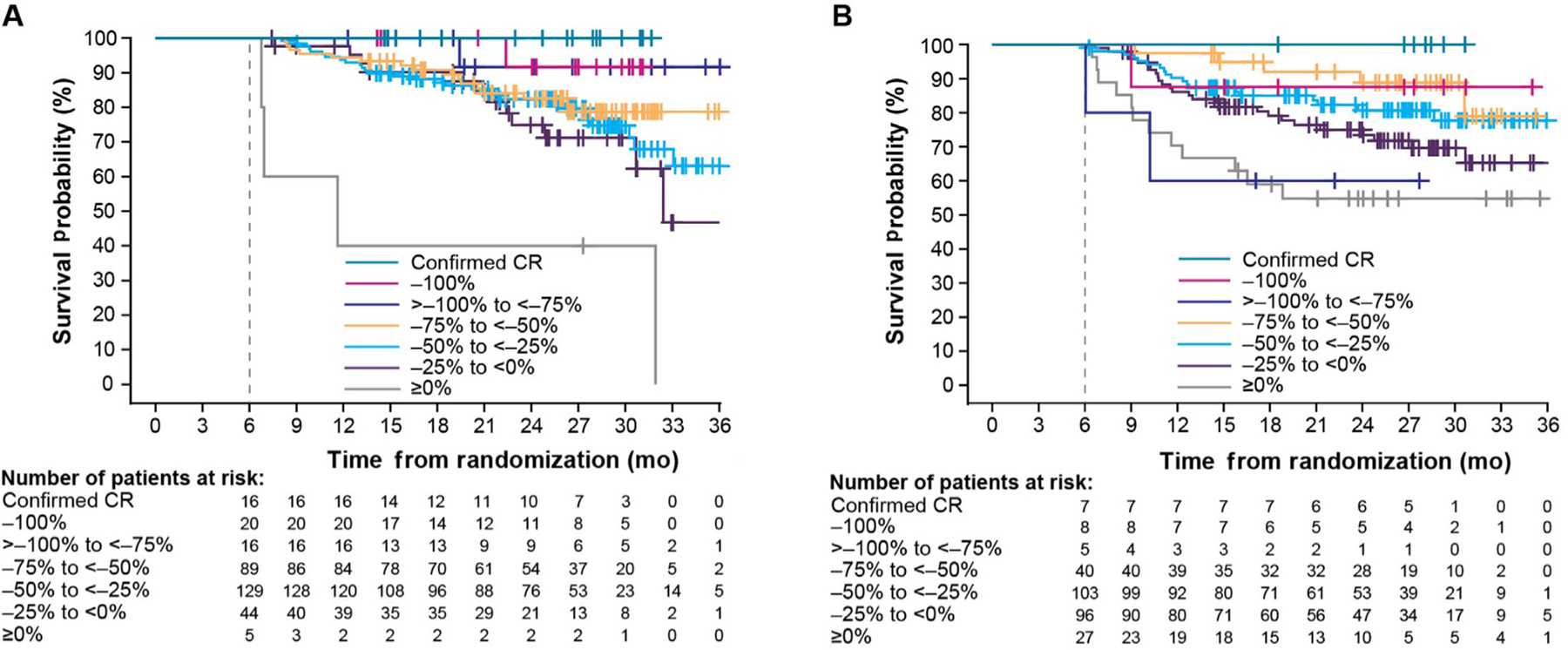
Six-month landmark analysis of overall survival by depth of response using RECIST v1.1 as per the independent review committee for the (A) lenvatinib plus pembrolizumab and (B) sunitinib treatment arms.+ = censored observations; CR = complete response; RECIST v1.1 = Response Evaluation Criteria in Solid Tumors version 1.1.

In the 6-mo landmark analysis of OS by tumor reduction, 12.4% (*n* = 44) of patients in the lenvatinib plus pembrolizumab arm and 4.5% (*n* = 16) in the sunitinib arm had achieved a confirmed CR or >75% tumor shrinkage by 6 mo. The OS probabilities at 24 mo were 100% (95% confidence interval [CI] not estimable [NE]-NE) among patients with a confirmed CR by 6 mo in the lenvatinib plus pembrolizumab arm (Fig. 1) and 91.7% (95% CI 53.9–98.8%) for patients with target-lesion reductions of both >75–<100% and 100% by 6 mo. In the sunitinib arm, the 6-mo landmark analysis showed that the OS probability at 24 mo was 100% (95% CI NE-NE) for patients with a confirmed CR, 87.5% (95% CI 38.7–98.1%) for patients with 100% target-lesion reduction, and 60.0% (95% CI 12.6–88.2%) for patients with >75–<100% target-lesion reduction. However, it should be noted that there were small numbers of patients with no tumor shrinkage in the lenvatinib plus pembrolizumab arm (*n* = 5) and patients with >75% tumor shrinkage in the sunitinib arm (*n* = 13) by 6 mo (Fig. 1), thereby limiting assessments in these small subgroups.

In the 9-mo landmark analyses of OS by tumor reduction, 18.0% (*n* = 64) of patients in the lenvatinib plus pembrolizumab arm and 5.3% (*n* = 19) in the sunitinib arm had achieved a confirmed CR or >75% tumor shrinkage at 9 mo. Results observed in the lenvatinib plus pembrolizumab arm were similar to the 6-mo landmark analysis (Supplementary Fig. 1). The 9-mo landmark analysis in the sunitinib arm was challenging to assess given the low patient numbers, particularly among patients with >75–<100% shrinkage (*n* = 3; Supplementary Fig. 1). The 6-mo landmark analysis of OS by disease progression is described in the Supplementary material and summarized in Supplementary Tables 2 and 3.

### 3.3. Efficacy results among the IMDC risk subgroups

**Fig. 2 –:**
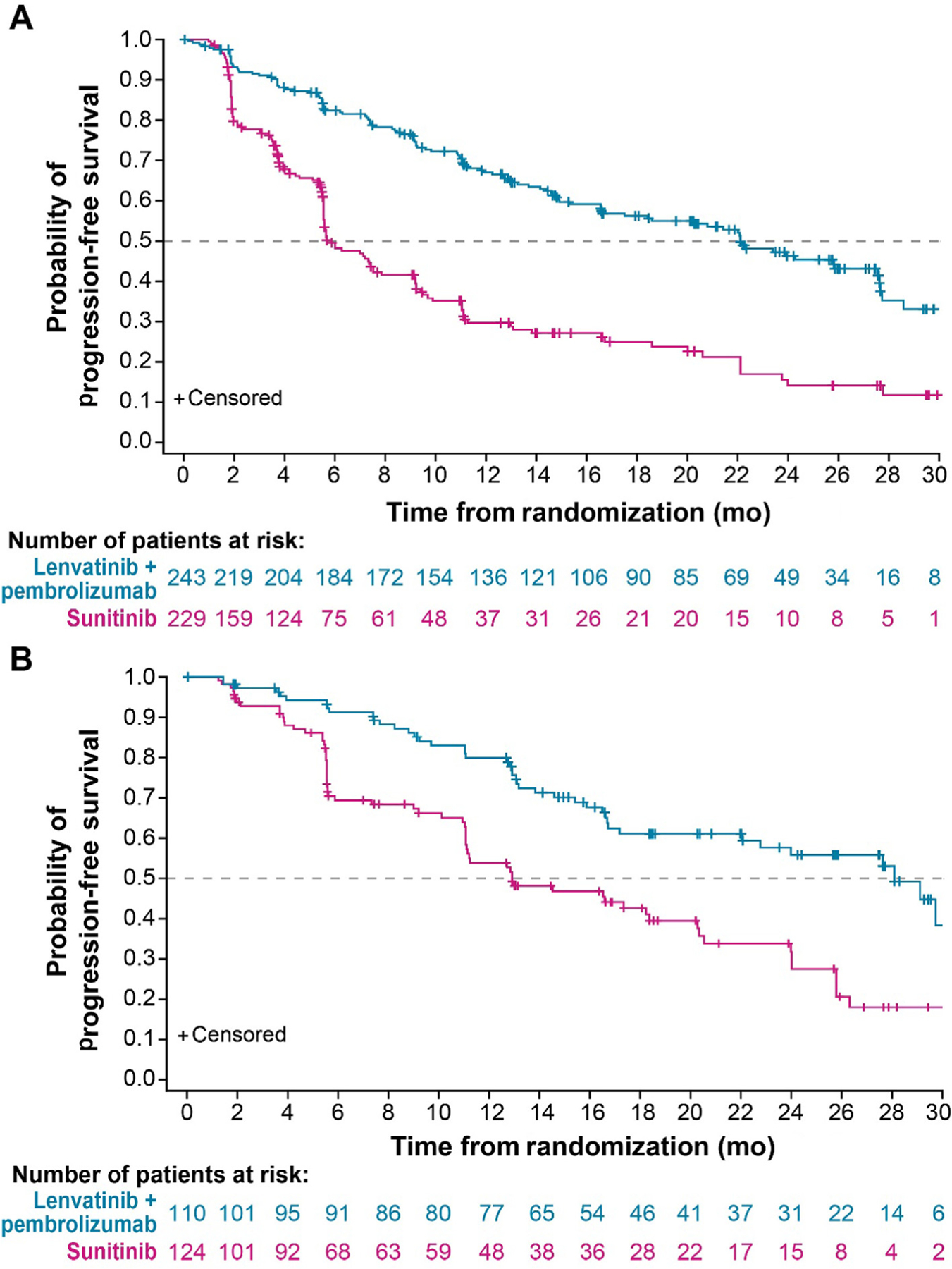
Kaplan-Meier plots of progression-free survival by the independent review committee as per RECIST v1.1 for the (A) IMDC intermediate/poor-risk and (B) IMDC favorable-risk subgroups. The IMDC risk score could not be evaluated for *n* = 2 and *n* = 4 patients in the lenvatinib plus pembrolizumab and sunitinib treatment arms, respectively.IMDC = International Metastatic Renal Cell Carcinoma Database Consortium; RECIST v1.1 = Response Evaluation Criteria in Solid Tumors version 1.1.

PFS results favored lenvatinib plus pembrolizumab versus sunitinib among patients in the IMDC intermediate/poor-risk subgroup (median PFS 22.1 [95% CI 16.6–27.6] vs 5.9 [95% CI 5.6–7.5] mo; hazard ratio [HR] 0.36, 95% CI 0.28–0.47) and in the IMDC favorable-risk subgroup (median PFS 28.1 [95% CI 22.0–NE] vs 12.9 [95% CI 11.1–18.4] mo; HR 0.41, 95% CI 0.28–0.62; Fig. 2 and Supplementary Table 4) [9]. Similar results were observed in the IMDC intermediate- and poor-risk subgroups individually (Supplementary Table 4) [9]. OS results favored lenvatinib plus pembrolizumab versus sunitinib treatment among patients in the IMDC intermediate/poor-risk subgroup (median not reached [NR] for both treatments; HR 0.58, 95% CI 0.42–0.80). In the IMDC favorable-risk subgroup (median NR for both treatments; HR 1.15, 95% CI 0.55–2.40), the low numbers of events observed (lenvatinib plus pembrolizumab arm, 14 deaths; sunitinib arm, 15 deaths) were considered inadequate to evaluate OS (Supplementary Table 4) [9]. In both the IMDC intermediate-risk and the IMDC poor-risk subgroup, OS favored lenvatinib plus pembrolizumab versus sunitinib treatment (Supplementary Table 4) [9].

ORR results favored lenvatinib plus pembrolizumab versus sunitinib treatment in the IMDC intermediate/poor-risk subgroup (72.4% vs 28.8%; odds ratio 6.60, 95% CI 4.39– 9.90) and the IMDC favorable-risk subgroup (68.2% vs 50.8%; odds ratio 2.00, 95% CI 1.17–3.42; Table 1). CRs with lenvatinib plus pembrolizumab were achieved in 14.0% and 20.9% of patients in the IMDC intermediate/poor- and favorable-risk subgroups, respectively, compared with 3.9% and 4.8% of patients, respectively, with sunitinib. CR rates were higher in the lenvatinib plus pembrolizumab arm than in the sunitinib arm, irrespective of the IMDC risk subgroup (Table 1). ORR results also favored lenvatinib plus pembrolizumab versus sunitinib treatment among patients in the IMDC intermediate-risk subgroup (72.9% vs 31.8%; odds ratio 6.01, 95% CI 3.88–9.32) and the IMDC poor-risk subgroup (69.7% vs 13.5%; odds ratio 11.19, 95% CI 3.37–37.15), separately (Table 1).

Tumor shrinkage was observed across IMDC risk subgroups in both treatment arms (Supplementary Fig. 2). Overall, evaluable patients in the lenvatinib plus pembrolizumab arm had a greater degree of tumor shrinkage than those in the sunitinib arm (≥50% reduction: 61.9% and 27.4%, respectively; Supplementary Fig. 2). In the IMDC favorable-risk subgroup, 71.3% of evaluable patients treated with lenvatinib plus pembrolizumab showed a reduction of ≥50% in target lesion size versus 37.7% of patients treated with sunitinib. In the IMDC intermediate-risk subgroup, ≥50% reduction in target lesions was observed in 59.5% of evaluable patients treated with lenvatinib plus pembrolizumab versus 22.4% of patients treated with sunitinib. In the IMDC poor-risk subgroup, 51.6% of evaluable patients treated with lenvatinib plus pembrolizumab showed a reduction of ≥50% in target lesions versus 19.2% of patients treated with sunitinib.

Similarly, the median percentage of target lesion shrinkage was greater in evaluable patients in the lenvatinib plus pembrolizumab arm (57.3%) than in the sunitinib arm (32.5%). A similar trend was observed across risk subgroups (IMDC favorable risk: 60.8% vs 40.5%; IMDC intermediate risk: 56.3% vs 31.1%; IMDC poor risk: 50.9% vs 17.9%).

### 3.4. Efficacy by presence or absence of target kidney lesions at baseline

**Fig. 3 –:**
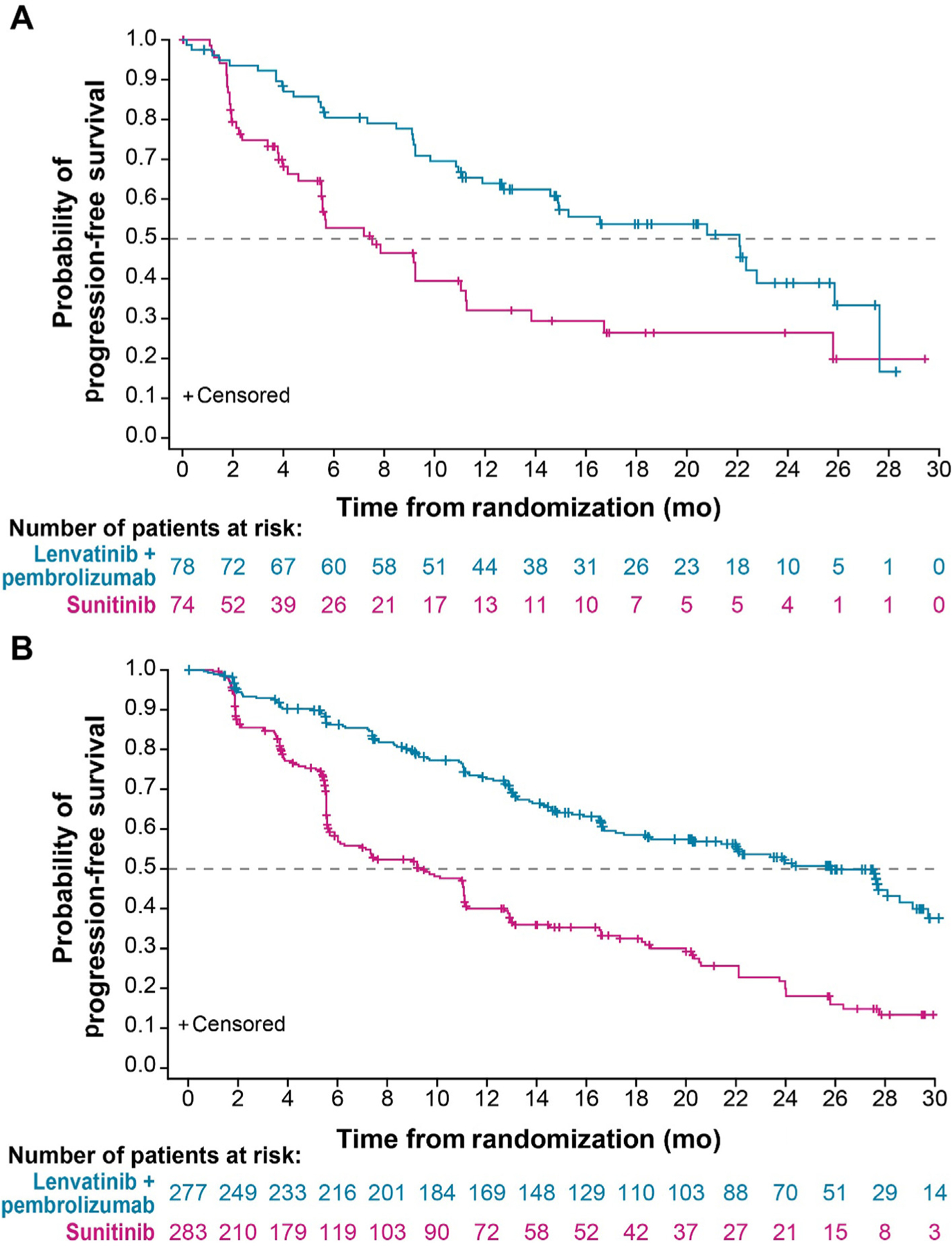
Kaplan-Meier plots of progression-free survival by the independent review committee as per RECIST v1.1 in patients (A) with and (B) without target kidney lesions.RECIST v1.1 = Response Evaluation Criteria in Solid Tumors version 1.1.

In patients with target kidney lesions at baseline, PFS (median 22.1 [95% CI 14.6–25.9] vs 7.5 [95% CI 5.5–11.2] mo; HR 0.40, 95% CI 0.25–0.65; Fig. 3 and Supplementary Table 5), OS (median NR vs 30.7 mo; HR 0.44, 95% CI 0.26–0.77), and ORR (71.8% vs 27.0%; odds ratio 10.55, 95% CI 4.54–24.52; Supplementary Table 5) all favored treatment with lenvatinib plus pembrolizumab versus sunitinib. Similar PFS (median 25.8 vs 9.4 mo, HR 0.38, 95% CI 0.30–0.49), OS (median NR vs NR, HR 0.76, 95% CI 0.54–1.09), and ORR (70.8% vs 38.5%; odds ratio 3.78, 95% CI 2.66–5.37) results were observed for lenvatinib plus pembrolizumab to sunitinib results observed among patients without target kidney lesions (Fig. 3 and Supplementary Table 5). When evaluating the overall shrinkage of target lesions, the median percentage of shrinkage was greater in the lenvatinib plus pembrolizumab arm than in the sunitinib arm for evaluable patients with baseline target kidney lesions (45.8% vs 19.6%) and for those without target kidney lesions (61.2% vs 36.4%).

Among those with target kidney lesions and no prior nephrectomy in the lenvatinib plus pembrolizumab arm, 56 evaluable patients were analyzed for tumor size reduction from baseline, of whom 100% showed a reduction of any size and 21.4% (*n* = 12) showed a reduction of ≥50% in target kidney lesions (Supplementary Fig. 3). Of the 43 evaluable patients analyzed in the sunitinib arm, 88.4% (*n* = 38) showed any reduction and 7.0% showed ≥50% reduction in target kidney lesions.

### 3.5. Characterizations of patients with a near CR

Overall, 114 (32.1%) patients in lenvatinib plus pembrolizumab arm and 41 (11.5%) in the sunitinib arm achieved a confirmed CR or a near CR (>75% shrinkage in target lesions). CRs and near CRs were observed across various subgroups, including IMDC risk groups, PD-L1 combined positive score, tumor stage, and lesion organ/sites in the lenvatinib plus pembrolizumab arm (Supplementary Table 6). In the sunitinib arm, a smaller proportion of patients with IMDC intermediate-risk (8.9%) or poor-risk (5.4%) disease achieved a confirmed CR or a near CR than those who had IMDC favorable-risk disease (17.7%); this trend was also seen in patients with initial tumor stages of II (4.8%), III (10.4%), and IV (8.7%) versus those with stage I (25.7%).

Among patients with a confirmed CR or near CR, the median DOR (95% CI) was NR (26.3–NE) in lenvatinib plus pembrolizumab arm and 24.0 mo (18.4–NE) in sunitinib arm, and the proportion of patients receiving any subsequent systemic anticancer therapy during survival followup was lower with lenvatinib plus pembrolizumab than with sunitinib (18.4% vs 36.6%; Supplementary Table 7).

## 4. Discussion

The results of this exploratory analysis support the primary findings of the pivotal phase 3 CLEAR trial [9]. Patients’ depth of response was positively associated with OS, particularly among those who achieved >75% tumor reduction within the first 6 mo of treatment. Additionally, lenvatinib plus pembrolizumab improved PFS and ORR regardless of IMDC risk subgroup and in the presence/absence of target kidney lesions. OS was also improved in the IMDC intermediate- and poor-risk subgroups and in the presence/absence of target kidney lesions, but OS data were too immature to confidently assess in the IMDC favorable subgroup.

Overall, tumor response was associated with OS irrespective of treatment. In particular, patients treated with lenvatinib plus pembrolizumab who had a deep tumor response (ie, tumor shrinkage of >75%) by 6 mo derived a similar survival probability over time versus those with a CR by 6 mo. At the 6-mo landmark, more than double the number of patients in the lenvatinib plus pembrolizumab arm had a confirmed CR or >75% reduction in the size of target lesions than that in the sunitinib arm, thereby highlighting the efficacy of lenvatinib plus pembrolizumab. Importantly, in the lenvatinib plus pembrolizumab arm, the rates at which patients achieved a CR or a >75% reduction were generally similar regardless of race, age, gender, IMDC risk subgroup, presence of metastases, or tumor stage at diagnosis. In the 6-mo landmark analysis of OS by disease progression, fewer patients in the lenvatinib plus pembrolizumab arm had progressive disease than in the sunitinib arm.

The results of this study are consistent with those reported previously. Specifically, a 6-mo landmark analysis of 2749 patients with metastatic RCC, who received sunitinib, sorafenib, temsirolimus, temsirolimus plus interferon alpha, or interferon alpha, demonstrated that the degree of patients’ tumor shrinkage was differentially associated with improved survival [21]; these benefits were also seen in a study of a tyrosine kinase inhibitor plus an ICI [23]. A similar association for RECIST-defined responses and OS with a 6-mo landmark analysis was reported in the CM214 trial of ipilimumab plus nivolumab [24].

Overall, the median PFS among the pooled IMDC intermediate- and poor-risk subgroups was favorable with lenvatinib plus pembrolizumab treatment (22.1 mo) versus sunitinib (5.9 mo). Other studies of ICI combination therapies in a similar population have reported median PFS ranging from 11.1 to 13.8 mo [8,25,26]. At 28.1 mo, the median PFS in the IMDC favorable-risk subgroup was particularly long in the lenvatinib plus pembrolizumab arm.

Although OS data in the IMDC favorable-risk subgroup are immature, it is notable that the lenvatinib plus pembrolizumab arm had a CR rate of 20.9% in this subgroup, and multiple patients in this subgroup had tumor shrinkage of >75% (Supplementary Fig. 2). Additionally, the IMDC favorable-risk subgroup of the sunitinib arm had a CR rate of only 4.8%. Considering the relation of tumor shrinkage with OS seen in the intention-to-treat population, these data suggest that lenvatinib plus pembrolizumab may provide an OS benefit in the IMDC favorable-risk subgroup; however, additional follow-up will be required to confirm this benefit. The importance of long-term follow-up can be seen based on the extended follow-up of the CM214 study of ipilimumab plus nivolumab, which noted a 48-mo survival probability of around 65.1% in patients with IMDC favorable-risk disease, compared with 50% in patients with IMDC intermediate/poor-risk disease [27]. As many of the events in that study occurred late (past the 48-mo time point) [27], additional follow-up of this study is required to confirm benefit.

It is important to note that this study has some notable limitations: it consisted of exploratory post hoc analyses and, thus, was not powered to detect significant differences between treatment groups. Additionally, there may not have been a sufficient number of events to evaluate OS by all subgroups, as the median OS for the lenvatinib plus pembrolizumab arm was NR in the intention-to-treat population [9].

Despite these limitations, this analysis of tumor dynamics as a predictive factor of outcome is valuable owing to the lack of long-term studies evaluating survival in patients with RCC treated with ICI-based combinations. While durable CRs with treatment for RCC have been reported in a small subset of patients after cytokine immunotherapy [28], 5-yr survival has historically been low—around 10– 20% [29]. Although ICI combination therapies have shown initial survival benefits for advanced/metastatic RCC [7– 9,11], long-term survival data are still limited. Notably, tumor shrinkage has been used to predict long-term survival for non-ICI therapies in patients with metastatic RCC [21]; a similar relationship between tumor shrinkage and survival has been suggested in a post hoc analysis of an ICI combination study [23]. The promising degree of tumor shrinkage with lenvatinib plus pembrolizumab suggests long-term survival benefits. However, continued analyses with extended follow-up are needed to confirm long-term survival benefits of lenvatinib plus pembrolizumab and other ICI combination therapies.

## 5. Conclusions

In this analysis, a greater percentage of patients assigned to lenvatinib plus pembrolizumab treatment had tumor shrinkage versus those treated with sunitinib. As this outcome appears to be related to OS, this analysis showcases the robust efficacy and long-term benefits for patients with advanced RCC treated first line with the combination of lenvatinib plus pembrolizumab.

Some information in this manuscript was presented at the American Society of Clinical Oncology Annual Meeting held on June 4–8, 2021.

## Supplementary Material

1

## Figures and Tables

**Table 1 –:** Summary of tumor response by IMDC risk subgroups using RECIST v1.1 per IRC

Parameter	IMDC intermediate/poor risk^a^		IMDC favorable risk^a^		IMDC intermediate risk^a^		IMDC poor risk^a^		Intention-to-treat population [9]	
	Lenvatinib + pembrolizumab (*n* = 243)	Sunitinib (*n* = 229)	Lenvatinib + pembrolizumab (*n* = 110)	Sunitinib (*n* = 124)	Lenvatinib + pembrolizumab (*n* = 210)	Sunitinib (*n* = 192)	Lenvatinib + pembrolizumab (*n* = 33)	Sunitinib (n = 37)	Lenvatinib + pembrolizumab (*n* = 355)	Sunitinib (*n* = 357)
BOR, *n* (%)										
CR	34 (14.0)	9 (3.9)	23 (20.9)	6 (4.8)	32 (15.2)	9 (4.7)	2 (6.1)	0	57 (16.1)	15 (4.2)
PR	142 (58.4)	57 (24.9)	52 (47.3)	57 (46.0)	121 (57.6)	52 (27.1)	21 (63.6)	5 (13.5)	195 (54.9)	114 (31.9)
SD	40 (16.5)	93 (40.6)	28 (25.5)	41 (33.1)	34 (16.2)	78 (40.6)	6 (18.2)	15 (40.5)	68 (19.2)	136 (38.1)
PD	15 (6.2)	42 (18.3)	3 (2.7)	8 (6.5)	12 (5.7)	33 (17.2)	3 (9.1)	9 (24.3)	19 (5.4)	50 (14.0)
Unknown/not evaluable	12 (4.9)	28 (12.2)	4 (3.6)	12 (9.7)	11 (5.2)	20 (10.4)	1 (3.0)	8 (21.6)	16 (4.5)	42 (11.8)
ORR (CR + PR), *n* (%)	176 (72.4)	66 (28.8)	75 (68.2)	63 (50.8)	153 (72.9)	61 (31.8)	23 (69.7)	5 (13.5)	252 (71.0)	129 (36.1)
95% CI^b^	66.8–78.0	23.0–34.7	59.5–76.9	42.0–59.6	66.8–78.9	25.2–38.4	54.0–85.4	2.5–24.5	66.3–75.7	31.2–41.1
Lenvatinib + pembrolizumab vs sunitinib										
Difference, % (95% CI)	43.6 (35.5–51.7)	–	17.4 (5.0–29.8)	–	41.1 (32.2–50.0)	–	56.2 (37.0–75.3)	–	34.9 (28.0–41.7)	–
Odds ratio^c^ (95% CI)	6.60 (4.39–9.90)	–	2.00 (1.17–3.42)	–	6.01 (3.88–9.32)	–	11.19 (3.37–37.15)	–	4.35 (3.16–5.97)	–
Median duration of response (mo)	25.8	12.9	26.3	14.7	25.9	12.9	20.4	NR	25.8	14.6
95% CI^d^	20.3–27.2	8.0–18.4	25.5–NE	9.3–19.0	20.2–NE	9.1–18.4	9.8–NE	2.6–NE	22.1–27.9	9.4–16.7
Patients with ongoing response at data cut in those with a CR, *n* (%)	29 (85.3)	5 (55.6)	18 (78.3)	3 (50.0)	27 (84.4)	5 (55.6)	2 (100)	0	47 (82.5)	8 (53.3)

BOR = best overall response; CI = confidence interval; CR = complete response; IMDC = International Metastatic Renal Cell Carcinoma Database Consortium; IRC = independent review committee; NE = not estimable; NR = not reached; ORR = objective response rate; PD = progressive disease; PR = partial response; RECIST v1.1 = Response Evaluation Criteria in Solid Tumors version 1.1; SD = stable disease.

a The IMDC risk score could not be evaluated for *n* = 2 and *n* = 4 patients in the lenvatinib plus pembrolizumab and sunitinib treatment arms, respectively.

b 95% CI was constructed using the method of normal approximation.

c Odds ratio was calculated using the Cochran-Mantel-Haenszel method, using IxRS stratification factors.

d 95% CI was estimated with a generalized Brookmeyer and Crowley method.

## References

[1] National Cancer Institute. Surveillance, Epidemiology, and End Results Program (SEER). Cancer stat facts: kidney and renal pelvis cancer https://seer.cancer.gov/statfacts/html/kidrp.html.

[2] International Agency for Research on Cancer (IARC). GLOBOCAN 2020. Cancer Today. https://gco.iarc.fr/today/home.

[3] Padala SA, Barsouk A, Thandra KC, Epidemiology of renal cell carcinoma. World J Oncol 2020;11:79–87. 32494314 10.14740/wjon1279 PMC7239575

[4] Li P, Wong YN, Armstrong K, Survival among patients with advanced renal cell carcinoma in the pretargeted versus targeted therapy eras. Cancer Med 2016;5:169–81. 26645975 10.1002/cam4.574 PMC4735783

[5] Choueiri TK, Motzer RJ. Systemic therapy for metastatic renal-cell carcinoma. N Engl J Med 2017;376:354–66. 28121507 10.1056/NEJMra1601333

[6] Choueiri TK, Kaelin WG Jr. Targeting the HIF2-VEGF axis in renal cell carcinoma. Nat Med 2020;26:1519–30. 33020645 10.1038/s41591-020-1093-z

[7] Choueiri TK, Powles T, Burotto M, Nivolumab plus cabozantinib versus sunitinib for advanced renal-cell carcinoma. N Engl J Med 2021;384:829–41. 33657295 10.1056/NEJMoa2026982 PMC8436591

[8] Motzer RJ, Tannir NM, McDermott DF, Nivolumab plus ipilimumab versus sunitinib in advanced renal-cell carcinoma. N Engl J Med 2018;378:1277–90. 29562145 10.1056/NEJMoa1712126 PMC5972549

[9] Motzer R, Alekseev B, Rha SY, Lenvatinib plus pembrolizumab or everolimus for advanced renal cell carcinoma. N Engl J Med 2021;384:1289–300. 33616314 10.1056/NEJMoa2035716

[10] Motzer RJ, Penkov K, Haanen J, Avelumab plus axitinib versus sunitinib for advanced renal-cell carcinoma. N Engl J Med 2019;380: 1103–15. 30779531 10.1056/NEJMoa1816047 PMC6716603

[11] Rini BI, Plimack ER, Stus V, Pembrolizumab plus axitinib versus sunitinib for advanced renal-cell carcinoma. N Engl J Med 2019;380:1116–27. 30779529 10.1056/NEJMoa1816714

[12] Rassy E, Flippot R, Albiges L. Tyrosine kinase inhibitors and immunotherapy combinations in renal cell carcinoma. Ther Adv Med Oncol 2020;12:1758835920907504. 10.1177/1758835920907504 PMC7081462 32215057

[13] National Comprehensive Cancer Network clinical practice guidelines in oncology (NCCN Guidelines^®^). Kidney cancer. Version 3. 2022. http://www.nccn.org/professionals/physician_gls/PDF/kidney.pdf.

[14] Bedke J, Albiges L, Capitanio U, Updated European Association of Urology guidelines on renal cell carcinoma: nivolumab plus cabozantinib joins immune checkpoint inhibition combination therapies for treatment-naïve metastatic clear-cell renal cell carcinoma. Eur Urol 2021;79:339–42. 33357997 10.1016/j.eururo.2020.12.005

[15] Powles T, Albiges L, Bex A, ESMO clinical practice guideline update on the use of immunotherapy in early stage and advanced renal cell carcinoma. Ann Oncol 2021;32:1511–9. 34597799 10.1016/j.annonc.2021.09.014

[16] Motzer RJ, Hutson TE, Glen H, Lenvatinib, everolimus, and the combination in patients with metastatic renal cell carcinoma: a randomised, phase 2, open-label, multicentre trial. Lancet Oncol 2015;16:1473–82. 26482279 10.1016/S1470-2045(15)00290-9

[17] Pal SK, Puente J, Heng DYC, Phase II trial of lenvatinib (LEN) at two starting doses + everolimus (EVE) in patients (pts) with renal cell carcinoma (RCC): results by independent imaging review (IIR) and prior immune checkpoint inhibition (ICI) [abstract]. J Clin Oncol 2021;39(6 Suppl):307.

[18] Larkin JMG, Tykodi SS, Donskov F, First-line pembrolizumab (pembro) monotherapy in advanced clear cell renal cell carcinoma (ccRCC): updated follow-up for KEYNOTE-427 cohort A. Ann Oncol 2019;30(Suppl 5):v381–2, Abstract 949P.

[19] Lee J-L, Ziobro M, Gafanov R, KEYNOTE-427 cohort B: first-line pembrolizumab (pembro) monotherapy for advanced non-clear cell renal cell carcinoma (NCC-RCC). J Clin Oncol 2019;37(15 Suppl):4569.

[20] Lee CH, Shah AY, Rasco D, Lenvatinib plus pembrolizumab in patients with either treatment-naive or previously treated metastatic renal cell carcinoma (study 111/KEYNOTE-146): a phase 1b/2 study. Lancet Oncol 2021;22:946–58. 34143969 10.1016/S1470-2045(21)00241-2 PMC8316679

[21] Grünwald V, McKay RR, Krajewski KM, Depth of remission is a prognostic factor for survival in patients with metastatic renal cell carcinoma. Eur Urol 2015;67:952–8. 25577718 10.1016/j.eururo.2014.12.036 PMC4570832

[22] Motzer R, Porta C, Alekseev B, Health-related quality of life outcomes in patients with advanced renal cell carcinoma treated with lenvatinib plus pembrolizumab or everolimus versus sunitinib: a randomized phase 3 study (CLEAR trial). Lancet Oncol 2022;23: 768–80. 35489363 10.1016/S1470-2045(22)00212-1 PMC10284118

[23] Powles T, Plimack ER, Soulières D, Pembrolizumab plus axitinib versus sunitinib monotherapy as first-line treatment of advanced renal cell carcinoma (KEYNOTE-426): extended follow-up from a randomised, open-label, phase 3 trial. Lancet Oncol 2020;21: 1563–73. 33284113 10.1016/S1470-2045(20)30436-8

[24] Motzer RJ, Escudier B, McDermott DF, Survival outcomes and independent response assessment with nivolumab plus ipilimumab versus sunitinib in patients with advanced renal cell carcinoma: 42-month follow-up of a randomized phase 3 clinical trial. J Immunother Cancer 2020;8:e000891. 32661118 10.1136/jitc-2020-000891 PMC7359377

[25] Rini BI, Plimack ER, Stus V, Pembrolizumab (pembro) plus axitinib (axi) versus sunitinib as first-line therapy for advanced clear cell renal cell carcinoma (ccRCC): results from 42-month follow-up of KEYNOTE-426. J Clin Oncol 2021;39(15 Suppl):4500.

[26] Haanen JBAG, Larkin J, Choueiri TK, Efficacy of avelumabaxitinib (AAx) versus sunitinib (S) by IMDC risk group in advanced renal cell carcinoma (aRCC): extended follow-up results from JAVELIN Renal 101. J Clin Oncol 2021;39(15 Suppl):4574.

[27] Albiges L, Tannir NM, Burotto M, Nivolumab plus ipilimumab versus sunitinib for first-line treatment of advanced renal cell carcinoma: extended 4-year follow-up of the phase III CheckMate 214 trial. ESMO Open 2020;5:e001079. 33246931 10.1136/esmoopen-2020-001079 PMC7703447

[28] Leary A, Larkin JM, Pickering LM. Cytokine therapy for renal cell cancer: the evolving role of immunomodulation. Therapy 2011;8: 347–58.

[29] Fisher RI, Rosenberg SA, Fyfe G. Long-term survival update for high-dose recombinant interleukin-2 in patients with renal cell carcinoma. Cancer J Sci Am 2000;6(Suppl 1):S55–7. 10685660

