# Supplementary material for: Survival by Depth of Response and Efficacy by International Metastatic Renal Cell Carcinoma Database Consortium Subgroup with Lenvatinib Plus Pembrolizumab Versus Sunitinib in Advanced Renal Cell Carcinoma: Analysis of the Phase 3 Randomized CLEAR Study
Source: Eur Urol Oncol. Author manuscript; Available in PMC 2024 Feb 19. (PMC10875602; doi:10.1016/j.euo.2023.01.010)

**Supplementary materials**

**Supplementary Methods**

***Statistical Analyses***

For the estimation of difference in treatment effect between lenvatinib plus pembrolizumab versus sunitinib, odds ratios were calculated using the stratified Cochran–Mantel–Haenszel method; hazard ratios (HRs) were based on stratified Cox proportional hazards model, using IxRS stratification factors. The 95% confidence intervals (CI) for the ORR were constructed using the method of normal approximation; PFS, OS, and DOR were evaluated by Kaplan–Meier method, and the 95% CI for their probability and median were estimated using a Greenwood formula and/or generalized Brookmeyer and Crowley method. While patients were stratified by MSKCC risk group, efficacy results were evaluated by IMDC risk group, which were derived programmatically and evaluated per six factors: KPS, time from diagnosis to first randomization, hemoglobin concentration, neutrophil count, platelet count, and serum calcium.^1^ IMDC risk groups of “intermediate” and “poor” were pooled retrospectively. For the post hoc 6- and 9-month landmark analyses of tumor shrinkage and OS, patients alive at the landmark timepoint were grouped based on maximum tumor shrinkage from baseline or a confirmed CR up to the landmark timepoint. The post hoc 6-month landmark analyses of progressive disease and OS were performed based on progression status within the first 6 months of treatment. Patients who were alive at 6 months were included to minimize lead-time bias.^2^

**References**

1. Ko JJ, Xie W, Kroeger N, et al. The International Metastatic Renal Cell Carcinoma Database Consortium model as a prognostic tool in patients with metastatic renal cell carcinoma previously treated with first-line targeted therapy: a population-based study. *Lancet Oncol.* 2015;16(3):293-300.
2. Heng DY, Xie W, Bjarnason GA, et al. Progression-free survival as a predictor of overall survival in metastatic renal cell carcinoma treated with contemporary targeted therapy. *Cancer.* 2011;117(12):2637-2642.

**Supplementary Results**

***6-month Landmark Analysis of Overall Survival (OS) by Disease Progression***

In the 6-month landmark analysis of OS by disease progression, 276 patients in the lenvatinib plus pembrolizumab arm and 145 patients in the sunitinib arm were alive and had no progressive disease (PD) at 6 months (**Supplementary Table 2**); patients with no progression at 6 months had lower probabilities of death in both arms. Among the 645 patients who were alive at 6 months, 80 were censored from follow-up for progression status (censoring was based on progression-free survival [PFS]) within 6 months of randomization, including 21 in the lenvatinib plus pembrolizumab arm and 59 in the sunitinib arm (**Supplementary Table 3**). The primary reason for censoring was the start of a new anticancer treatment (55/80). A greater proportion and number of patients in the sunitinib arm (75%; 44/59) were censored for receiving new therapy than in the lenvatinib plus pembrolizumab arm (52%; 11/21).

**Supplementary Table 1 –** Baseline demographic and clinical characteristics of patients in the CLEAR trial (Study 307/KEYNOTE 581)^1^

| Parameter | Lenvatinib + pembrolizumab  (*n* = 355) | | Sunitinib  (*n* = 357) | |
| --- | --- | --- | --- | --- |
| Median age, years (range) | 64 (34–88) | | 61 (29–82) | |
| Geographic region, *n* (%) |  | |  | |
| Western Europe/North America | 198 (55.8) | | 199 (55.7) | |
| Rest of the world | 157 (44.2) | | 158 (44.3) | |
| Sex | | | | |
| Female | 100 (28.2) | | 82 (23.0) | |
| Male | 255 (71.8) | | 275 (77.0) | |
| Race | | | | |
| Asian | 81 (22.8) | | 67 (18.8) | |
| White | 263 (74.1) | | 270 (75.6) | |
| Others | 6 (1.7) | | 10 (2.8) | |
| Missing | 5 (1.4) | | 10 (2.8) | |
| MSKCC prognostic risk group, *n* (%) | | | | |
| Favorable | 96 (27.0) | | 97 (27.2) | |
| Intermediate | 227 (63.9) | | 228 (63.9) | |
| Poor | 32 (9.0) | | 32 (9.0) | |
| IMDC risk subgroup, *n* (%) |  | |  | |
| Favorable | 110 (31.0) | | 124 (34.7) | |
| Intermediate | 210 (59.2) | | 192 (53.8) | |
| Poor | 33 (9.3) | | 37 (10.4) | |
| Could not be evaluated | 2 (0.6) | | 4 (1.1) | |
| AJCC stage at diagnosis, *n* (%) | | | | |
| I | 50 (14.1) | | 35 (9.8) | |
| II | 16 (4.5) | | 21 (5.9) | |
| III | 60 (16.9) | | 67 (18.8) | |
| IV | 178 (50.1) | | 195 (54.6) | |
| Not assigned | 51 (14.4) | | 39 (10.9) | |
| Sarcomatoid features, *n* (%) | 28 (7.9) | | 21 (5.9) | |
| PD-L1 combined positive score, *n* (%) | | | | |
| ≥1 | 107 (30.1) | | 119 (33.3) | |
| <1 | 112 (31.5) | | 103 (28.9) | |
| Not available | 136 (38.3) | | 135 (37.8) | |
| Patients with target kidney lesions | | | | |
| Yes / no, *n* (%) | 78 (22.0) / 277 (78.0) | | 74 (20.7) / 283 (79.3) | |
| Median sum of diameter(s) of target kidney lesions, mm (range) | 80.7 (11.8, 175.7) | | 77.0 (19.0, 182.8) | |
| Number of target kidney lesions, *n* (%) | | | | |
| 0 | 277 (78.0) | | 283 (79.3) | |
| 1 | 67 (18.9) | | 59 (16.5) | |
| 2 | 11 (3.1) | | 15 (4.2) | |
| Median sum of diameter(s) of target kidney lesions by number of target kidney lesions, mm (range) | | | | |
| 1 | 79.7 (11.8–175.7) | | 76.5 (19.0–159.6) | |
| 2 | 85.2 (30.2–164.0) | | 97.6 (23.1–182.8) | |
| Prior nephrectomy, *n* (%) | 262 (73.8) | | 275 (77.0) | |
| Number of target lesions by primary nephrectomy status, *n* (%) | Nephrectomy (*n*=262) | No nephrectomy (*n*=93) | Nephrectomy  (*n*=275) | No nephrectomy  (*n*=82) |
| 0 | 242 (68.2) | 35 (9.9) | 253 (70.9) | 30 (8.4) |
| 1 | 14 (3.9) | 53 (14.9) | 16 (4.5) | 43 (12.0) |
| 2 | 6 (1.7) | 5 (1.4) | 6 (1.7) | 9 (2.5) |
| Number of metastatic organs/sites, *n* (%)^a^ | | | | |
| 1 | 119 (33.5) | | 114 (31.9) | |
| 2 | 129 (36.3) | | 127 (35.6) | |
| ≥3 | 102 (28.7) | | 109 (30.5) | |
| Lesion organ/site location, *n* (%)^a,b^ | | | | |
| Adrenal | 53 (14.9) | | 66 (18.5) | |
| Bone | 80 (22.5) | | 89 (24.9) | |
| Brain | 6 (1.7) | | 10 (2.8) | |
| Kidney | 91 (25.6) | | 88 (24.6) | |
| Liver | 63 (17.7) | | 70 (19.6) | |
| Lung | 252 (71.0) | | 228 (63.9) | |
| Lymph node | 162 (45.6) | | 156 (43.7) | |
| Other | 109 (30.7) | | 123 (34.5) | |

AJCC = American Joint Committee on Cancer; IMDC, =International Metastatic Renal Cell Carcinoma Database Consortium; MSKCC = Memorial Sloan Kettering Cancer Center; PD-L1 = programmed cell death ligand 1.

^a^Per independent review committee; ^b^patients could have been included in more than 1 category.

**Reference**

1. Motzer R, Alekseev B, Rha SY, et al. Lenvatinib plus pembrolizumab or everolimus for advanced renal cell carcinoma. N Engl J Med. 2021;384(14):1289-1300.

**Supplementary Table 2** – Summary of survival outcomes in the 6-month landmark analysis of overall survival by PD status

| Parameter | Lenvatinib + pembrolizumab | | Sunitinib | |
| --- | --- | --- | --- | --- |
|  | PD ≤6 months  (*n*=41) | No PD ≤6 months  (*n*=276) | PD ≤6 months  (*n*=103) | No PD ≤6 months  (*n*=145) |
| Probability of death^a^ at 12 mo, % (95% CI) | 15.0 (7.0–30.5) | 4.0 (2.2–7.1) | 27.2 (19.6–36.9) | 2.8 (1.1–7.3) |

CI = confidence interval; PD = progressive disease.

^a^Deaths were reported in patients who were alive at 6 months from randomization.

**Supplementary Table 3** – Patients’ status for overall survival, progression, and start of subsequent anticancer medication at 6 months

| Patients’ status at 6 months, *n* (%) | Lenvatinib + pembrolizumab  (*n*=355) | Sunitinib  (*n*=357) | Total  (*n*=712) |
| --- | --- | --- | --- |
| Alive at 6 months |  |  |  |
| No PD at 6 months | 276 (77.7) | 145 (40.6) | 421 (59.1) |
| Had PD and started subsequent anticancer medication within 6 months | 7 (2.0) | 39 (10.9) | 46 (6.5) |
| Had PD but did not receive subsequent anticancer medication within 6 months | 34 (9.6) | 64 (17.9) | 98 (13.8) |
| Had PFS censored within 6 months  Due to new anticancer treatment started^a^ | 21 (5.9)  11 (52.4) | 59 (16.5)  44 (74.6) | 80 (11.2)  55 (68.8) |
| Death within 6 months |  |  |  |
| No PD before death | 7 (2.0) | 7 (2.0) | 14 (2.0) |
| Had PD and started subsequent anticancer medication within 6 months | 0 (0.0) | 8 (2.2) | 8 (1.1) |
| Had PD but did not receive subsequent anticancer medication within 6 months | 2 (0.6) | 6 (1.7) | 8 (1.1) |
| Had PFS censored within 6 months  Due to new anticancer treatment started^a^ | 1 (0.3)  1 (100) | 7 (2.0)  7 (100) | 8 (1.1)  8 (100) |
| OS censored within 6 months |  |  |  |
| Had PD and started subsequent anticancer medication within 6 months | 0 (0.0) | 1 (0.3) | 1 (0.1) |
| Had PD but did not receive subsequent anticancer medication within 6 months | 1 (0.3) | 1 (0.3) | 2 (0.3) |
| Had PFS censored within 6 months  Due to new anticancer treatment started^a^ | 6 (1.7)  0 (0.0) | 20 (5.6)  0 (0.0) | 26 (3.7)  0 (0.0) |

PD = progressive disease; PFS = progression-free survival; OS = overall survival.

^a^Percentages are based on the number of patients who were censored for progression-free survival within 6 months.

**Supplementary Table 4** – Summary of survival outcomes by IMDC risk subgroups

| Parameter | IMDC-intermediate/poor risk | | IMDC-favorable risk | | IMDC-intermediate risk | | IMDC-poor risk | |
| --- | --- | --- | --- | --- | --- | --- | --- | --- |
|  | Lenvatinib + pembrolizumab (*n*=243) | Sunitinib (*n*=229) | Lenvatinib + pembrolizumab (*n*=110) | Sunitinib (*n*=124) | Lenvatinib + pembrolizumab (*n*=210) | Sunitinib  (*n*=192) | Lenvatinib + pembrolizumab (*n*=33) | Sunitinib  (*n*=37) |
| Progression-free survival^a^ |  |  |  |  |  |  |  |  |
| Median, months | 22.1 | 5.9 | 28.1 | 12.9 | 22.1 | 7.1 | 22.1 | 4.0 |
| Hazard ratio for lenvatinib + pembrolizumab vs sunitinib | 0.36 | - | 0.41^1^ | - | 0.39^2^ | - | 0.28^1^ | - |
| 95% CI | 0.28–0.47 | - | 0.28–0.62^1^ | - | 0.29–0.52^2^ | - | 0.13–0.60^1^ | - |
| Overall survival |  |  |  |  |  |  |  |  |
| Median, months | NR | NR | NR | NR | NR | NR | NR | 10.4 |
| Hazard ratio for lenvatinib + pembrolizumab vs sunitinib | 0.58 | - | 1.15^1^ | - | 0.72^1^ | - | 0.30^1^ | - |
| 95% CI | 0.42–0.80 | - | 0.55–2.40^1^ | - | 0.50–1.05^1^ | - | 0.14–0.64^1^ | - |

CI = confidence interval; IMDC = International Metastatic Renal Cell Carcinoma Database Consortium; NR = not reached; RECIST v1.1 = Response Evaluation Criteria In Solid Tumors version 1.1.

^a^Per RECIST v1.1 by independent review committee.

**References**

1. Motzer R, Alekseev B, Rha SY, et al. Lenvatinib plus pembrolizumab or everolimus for advanced renal cell carcinoma. N Engl J Med. 2021;384(14):1289-1300.

2. Ko JJ, Xie W, Kroeger N, et al. The International Metastatic Renal Cell Carcinoma Database Consortium model as a prognostic tool in patients with metastatic renal cell carcinoma previously treated with first-line targeted therapy: a population-based study. Lancet Oncol. 2015;16(3):293-300.

**Supplementary Table 5** – Summary of tumor response by target kidney lesion subgroups using RECIST v1.1 per IRC

| Parameter | Patients with target kidney lesions | | Patients without target kidney lesions | |
| --- | --- | --- | --- | --- |
|  | Lenvatinib + pembrolizumab  (*n*=78) | Sunitinib  (*n*=74) | Lenvatinib + pembrolizumab (*n*=277) | Sunitinib  (*n*=283) |
| Best overall response, *n* (%) |  |  |  |  |
| CR | 2 (2.6) | 1 (1.4) | 55 (19.9) | 14 (4.9) |
| PR | 54 (69.2) | 19 (25.7) | 141 (50.9) | 95 (33.6) |
| Stable disease | 13 (16.7) | 31 (41.9) | 55 (19.9) | 105 (37.1) |
| Progressive disease | 4 (5.1) | 14 (18.9) | 15 (5.4) | 36 (12.7) |
| Unknown/Not evaluable | 5 (6.4) | 9 (12.2) | 11 (4.0) | 33 (11.7) |
| Objective response rate (CR + PR), *n* (%) | 56 (71.8) | 20 (27.0) | 196 (70.8) | 109 (38.5) |
| 95% CI^a^ | 61.8–81.8 | 16.9–37.1 | 65.4–76.1 | 32.8–44.2 |
| Lenvatinib + pembrolizumab vs sunitinib |  |  |  |  |
| Difference, % (95% CI)^a^ | 44.8 (30.6–59.0) | - | 32.2 (24.4–40.0) | - |
| Odds ratio^b^ (95% CI) | 10.55 (4.54–24.52) | - | 3.78 (2.66–5.37) | - |
| Median duration of response, months | 20.3 | 24.0 | 26.0 | 13.1 |
| 95% CI^c^ | 14.7–25.8 | 3.7–NE | 22.4–NE | 9.4–16.7 |
| Median progression-free survival, months^a^ (95% CI) | 22.1 (14.6–25.9) | 7.5 (5.5–11.2) | 25.8 (21.4–28.6) | 9.4 (6.1–11.1) |
| Hazard ratio (95% CI) | 0.40 (0.25–0.65) | - | 0.38 (0.30–0.49) | - |

CI = confidence interval; CR = complete response; IRC = independent review committee; NE = not estimable; PR = partial response; RECIST v1.1 = Response Evaluation Criteria In Solid Tumors version 1.1.

^a^95% CI constructed using the method of normal approximation.

^b^Odds ratio calculated using the Cochran–Mantel–Haenszel method, using IxRS stratification factors.

^c^95% CIs estimated with a generalized Brookmeyer and Crowley method.

**Supplementary Table 6** – Characterization of patients in the lenvatinib plus pembrolizumab arm who had a CR or >75% reduction in tumor size by baseline factors

| Baseline characteristic, *n*_1_/*n*^a^ (%) | Lenvatinib + pembrolizumab | Sunitinib |
| --- | --- | --- |
| All Patients | 114/355 (32.1) | 41/357 (11.5) |
| Region | |  |
| Western Europe and North America | 64/198 (32.3) | 28/199 (14.1) |
| Rest of the world | 50/157 (31.8) | 13/158 (8.2) |
| Age | |  |
| <65 years | 69/194 (35.6) | 23/225 (10.2) |
| ≥65 years | 45/161 (28.0) | 18/132 (13.6) |
| Sex | |  |
| Female | 26/100 (26.0) | 30/275 (10.9) |
| Male | 88/255 (34.5) | 11/82 (13.4) |
| Race | |  |
| Asian | 28/81 (34.6) | 8/67 (11.9) |
| White | 83/263 (31.6) | 30/270 (11.1) |
| IMDC risk subgroup | |  |
| Favorable | 40/110 (36.4) | 22/124 (17.7) |
| Intermediate | 66/210 (31.4) | 17/192 (8.9) |
| Poor | 7/33 (21.2) | 2/37 (5.4) |
| AJCC stage at diagnosis | |  |
| I | 19/50 (38.0) | 9/35 (25.7) |
| II | 7/16 (43.8) | 1/21 (4.8) |
| III | 31/60 (51.7) | 7/67 (10.4) |
| IV | 40/178 (22.5) | 17/195 (8.7) |
| Not assigned | 17/51 (33.3) | 7/39 (17.9) |
| Sarcomatoid features | 9/28 (32.1) | 0/21 (0) |
| PD-L1 combined positive score | |  |
| ≥1 (positive) | 42/107 (39.3) | 14/119 (11.8) |
| <1 (negative) | 34/112 (30.4) | 12/103 (11.7) |
| Not available | 38/136 (27.9) | 15/135 (11.1) |
| Prior nephrectomy | |  |
| Yes | 103/262 (39.3) | 39/275 (14.2) |
| No | 11/93 (11.8) | 2/82 (2.4) |
| Number of metastatic sites^b^ | |  |
| 1 | 51/119 (42.9) | 18/114 (15.8) |
| 2 | 40/129 (31.0) | 13/127 (10.2) |
| ≥3 | 23/102 (22.5) | 9/109 (8.3) |
| Lesion organ/site location, *n* (%)^b,c^ | |  |
| Adrenal | 16/53 (30.2) | 5/66 (7.6) |
| Bone | 19/80 (23.8) | 4/89 (4.5) |
| Brain | 1/6 (16.7) | 0/10 (0) |
| Kidney | 15/91 (16.5) | 5/88 (5.7) |
| Liver | 13/63 (20.6) | 3/70 (4.3) |
| Lung | 83/252 (32.9) | 23/228 (10.1) |
| Lymph node | 50/162 (30.9) | 20/156 (10.1) |
| Other | 27/109 (24.8) | 16/123 (13.0) |

AJCC = American Joint Committee on Cancer; CR = complete response; IMDC = International Metastatic Renal Cell Carcinoma Database Consortium; PD-L1 = programmed cell death ligand 1.

^a^Defined as the number of patients with a CR or tumor reduction of >75% during study (*n*_1_) over all patients (*n*); ^b^per independent review committee; ^c^patients could have been included in more than 1 category.

**Supplementary Table 7** – Summary of safety and efficacy results in patients who had a confirmed CR or >75% reduction in tumor size

| Parameter | Patients with a confirmed CR or >75% shrinkage in target lesions | |
| --- | --- | --- |
|  | Lenvatinib + pembrolizumab  (*n*=114) | Sunitinib  (*n*=41) |
| Treatment Ongoing at Cutoff Date, *n* (%) | 65 (57.0) | 21 (51.2) |
| Median duration of treatment, months (range) | 23.6 (2.8–38.9) | 19.9 (4.4–35.3) |
| Median time to first objective response, months (range)^a^ | 1.9 (1.5–17.3) | 1.9 (1.6–5.7) |
| Median duration of response, months  (95% CI)^a^ | NR (26.3–NE) | 24.0 (18.4–NE) |
| Patients receiving any subsequent systemic anticancer medications during survival  follow-up, *n* (%) | 21 (18.4) | 15 (36.6) |
| Patients with Any Treatment Related TEAEs with Worst Grade ≥3, *n* (%) | 85 (74.6) | 27 (65.9) |

CI = confidence interval; CR = complete response; NE = not estimable; NR = not reached; TEAE = treatment emergent adverse event.

^a^By independent review committee per Response Evaluation Criteria In Solid Tumors version 1.1.

**Supplementary Fig. 1 –** **Nine-month landmark analysis of overall survival by depth of response using RECIST v1.1 per independent review committee for patients in the lenvatinib plus pembrolizumab (A) and sunitinib (B) treatment arms. CR = complete response; RECIST v1.1 = Response Evaluation Criteria In Solid Tumors version 1.1.**


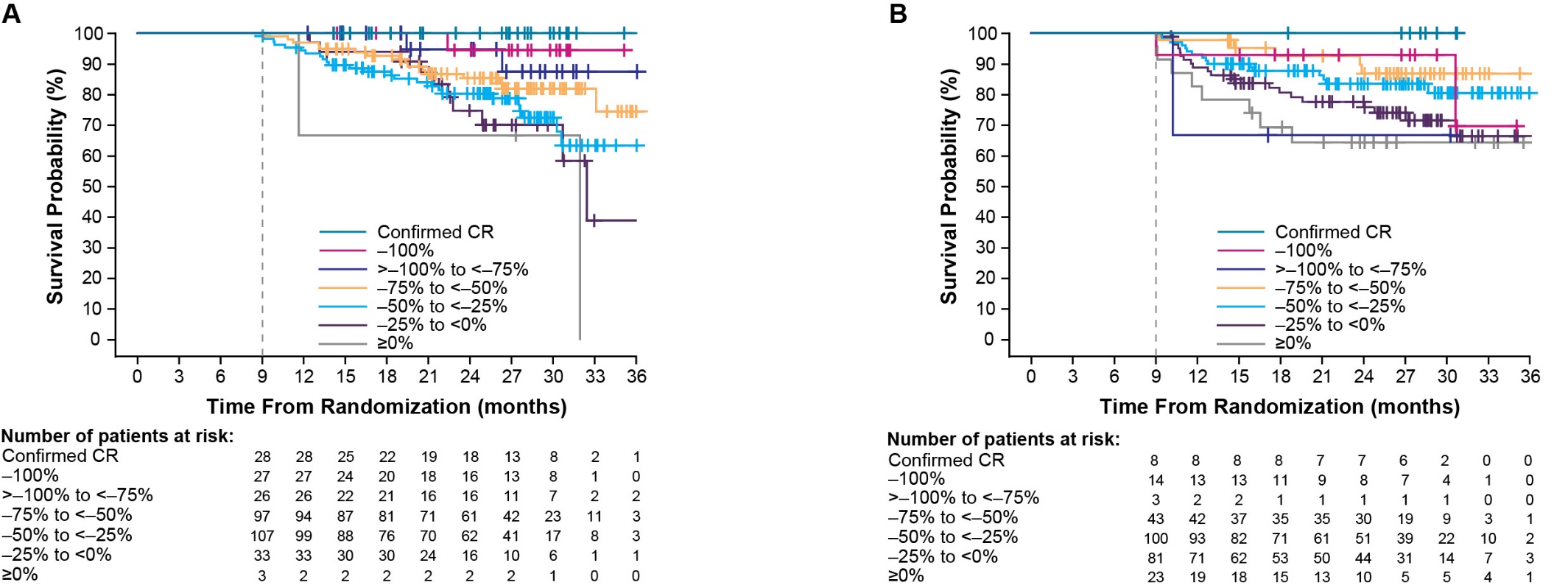


**Supplementary Fig. 2 – Maximum tumor shrinkage from baseline in target lesions by IMDC risk subgroup for patients in the (A) lenvatinib + pembrolizumab and (B) sunitinib arms using RECIST v1.1 by independent review committee. IMDC = International Metastatic Renal Cell Carcinoma Database Consortium; m = number of patients with both baseline and at least 1 postbaseline target lesion assessment; NE = not evaluable; RECIST v1.1 = Response Evaluation Criteria In Solid Tumors version 1.1.**

**
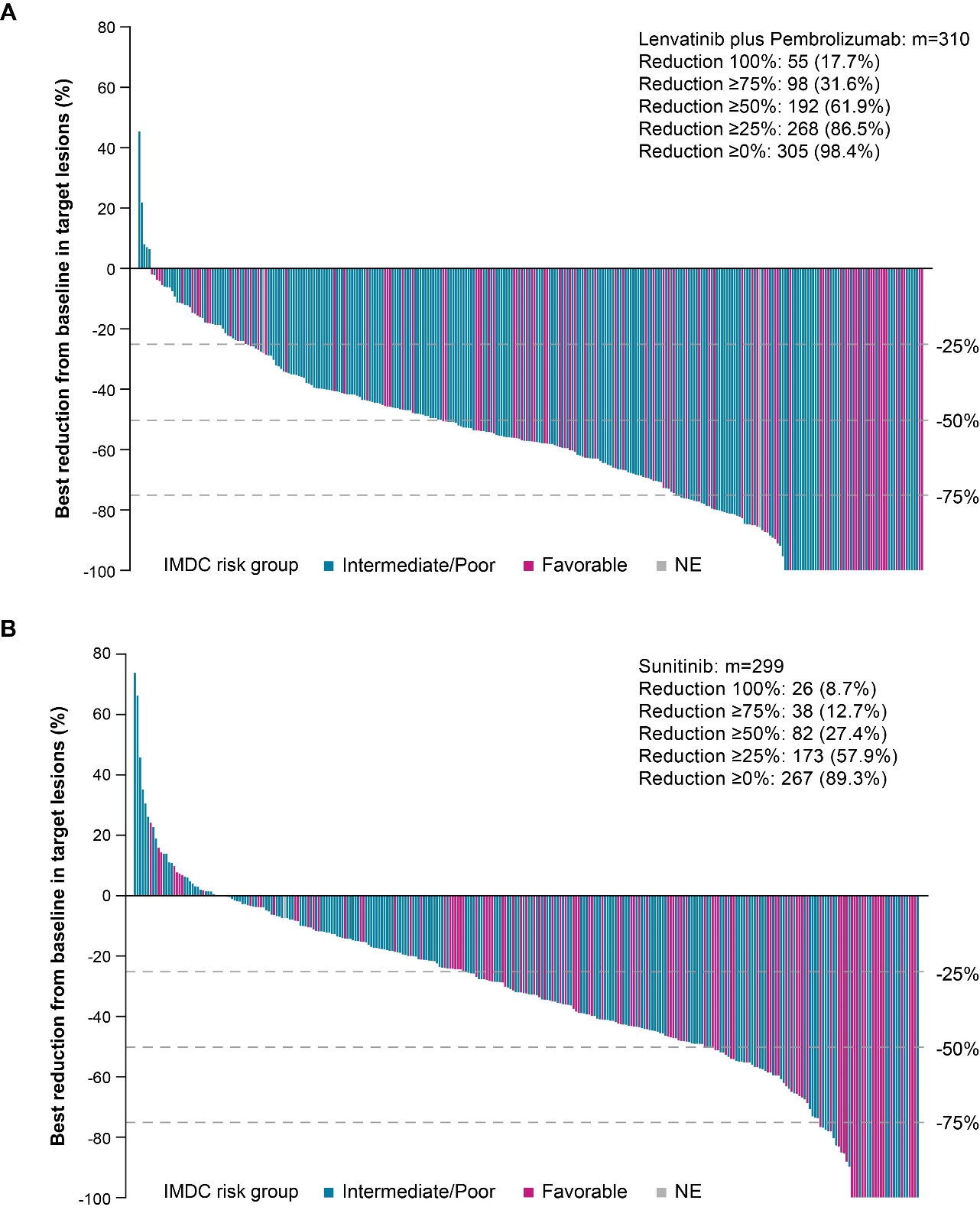
**

**Supplementary Fig. 3 –** **Maximum tumor shrinkage from baseline in target kidney lesions per independent review committee using RECIST v1.1 for patients without prior nephrectomy in the lenvatinib plus pembrolizumab (A) and sunitinib (B) treatment arms. m = number of patients with both baseline and at least 1 postbaseline target kidney lesion assessment; RECIST v1.1 = Response Evaluation Criteria In Solid Tumors version 1.1.**


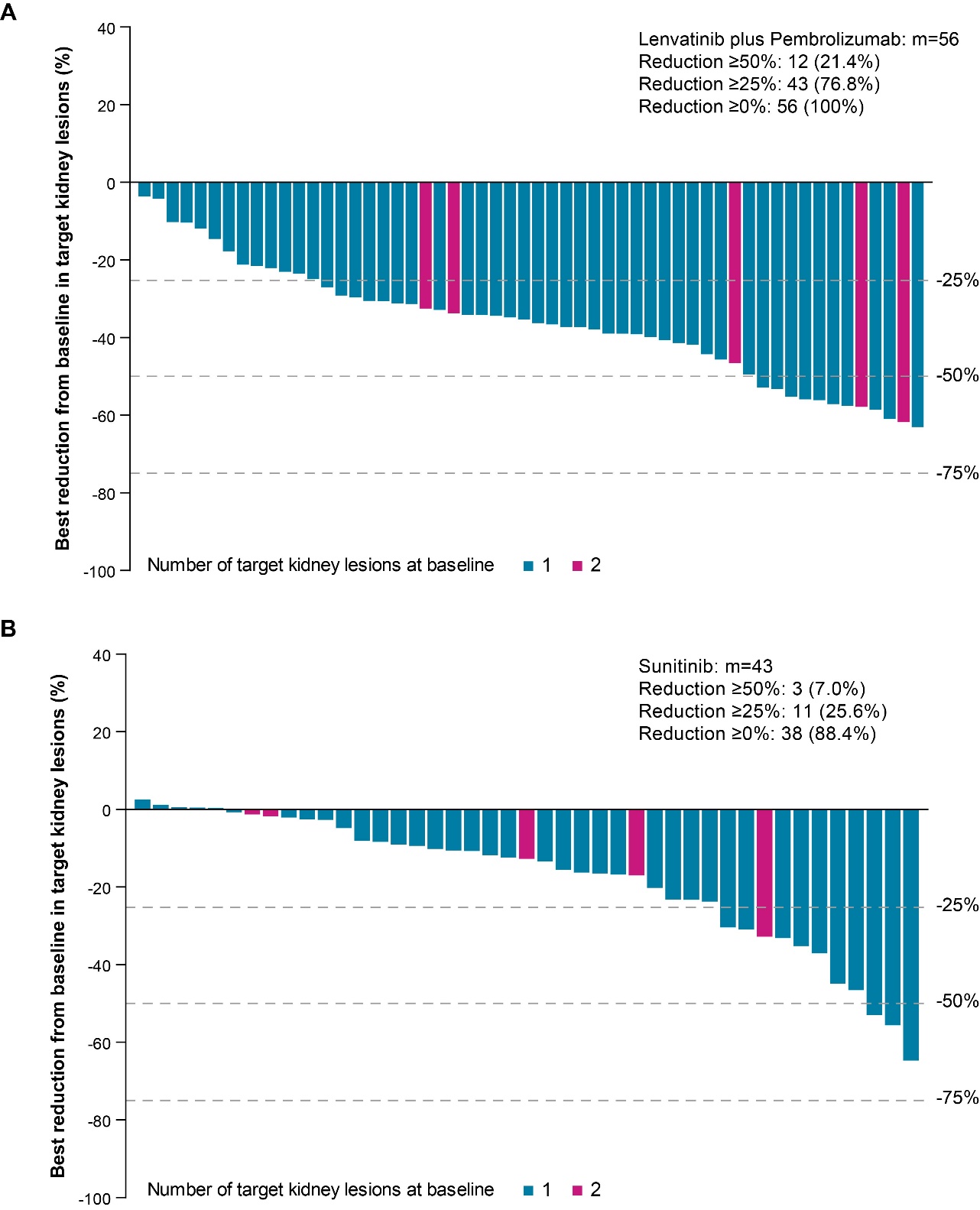

Supplement: 1 [file NIHMS1958004-supplement-1.docx]
